# Infection prevention measures for patients undergoing hemodialysis during the COVID-19 pandemic in Japan: a nationwide questionnaire survey

**DOI:** 10.1186/s41100-021-00350-y

**Published:** 2021-05-29

**Authors:** Yuka Sugawara, Masao Iwagami, Kan Kikuchi, Yoko Yoshida, Ryoichi Ando, Toshio Shinoda, Munekazu Ryuzaki, Hidetomo Nakamoto, Ken Sakai, Norio Hanafusa, Naoki Kashihara, Masaomi Nangaku, On behalf of COVID-19 Task Force Committee of the Japanese Association of Dialysis Physicians, the Japanese Society for Dialysis Therapy, and the Japanese Society of Nephrology

**Affiliations:** 1 Division of Nephrology and Endocrinology, The University of Tokyo, 7-3-1 Hongo, Bunkyo-ku, Tokyo, 113-8655 Japan; 2 Department of Health Services Research, Faculty of Medicine, University of Tsukuba, Ibaraki, Japan; 3 Faculty of Epidemiology and Population Health, London School of Hygiene and Tropical Medicine, London, UK; 4 Division of Nephrology, Shimoochiai Clinic, Tokyo, Japan; 5 Department of Nephrology, Seishokai Memorial Hospital, Tokyo, Japan; 6 Faculty of Medical and Health Sciences, Tsukuba International University, Tsuchiura, Japan; 7 Department of Nephrology, Tokyo Saiseikai Central Hospital, Tokyo, Japan; 8 Department of General Internal Medicine, Saitama Medical University, Saitama, Japan; 9 Department of Nephrology, Toho University Faculty of Medicine, Tokyo, Japan; 10 Department of Blood Purification, Tokyo Women’s Medical University, Tokyo, Japan; 11 Department of Nephrology and Hypertension, Kawasaki Medical School, Kurashiki, Japan

**Keywords:** COVID-19, SARS-CoV-2, Pandemic, Hemodialysis, Infection prevention measures, PPEs, Disinfectants, Isolation, Nosocomial transmission, Japan

## Abstract

**Background:**

Coronavirus disease 2019 (COVID-19), caused by severe acute respiratory syndrome coronavirus 2 (SARS-CoV-2), has become a global pandemic affecting a variety of medical treatments, including hemodialysis. This study aims to investigate the implementation of infection control measures, to examine the shortage of personal protective equipment (PPE) and disinfectants, and to quantify the number of nosocomial COVID-19 transmissions in hemodialysis facilities in Japan during the pandemic.

**Methods:**

We conducted a nationwide questionnaire survey between 20 October and 16 November 2020 (i.e., between the “second wave” and “third wave” in Japan) in the 4198 dialysis facilities of the Japanese Association of Dialysis Physicians and the Japanese Society for Dialysis Therapy. A total of 2227 facilities (53.0%) responded. The questionnaire consisted of (i) characteristics of facilities, (ii) infection prevention measures in routine dialysis practices, (iii) shortage of PPE, (iv) feasibility of various isolation measures, and (v) nosocomial transmission.

**Results:**

Half of the responding facilities were hospitals with multiple departments, and the other half were clinics specialized in dialysis. Several infection prevention measures such as health checks of staff and patients, donning of masks before and after hemodialysis, and disinfection of frequently contacted areas were implemented during the COVID-19 pandemic. There was a significant improvement in the implementation rate of these measures during the pandemic, compared to before it, which reached over 90%. More than half of the facilities reported a shortage of disposable masks (67.2%) and hand sanitizer alcohol (56.7%). Isolation of COVID-19 patients in private rooms was possible only in 52.7% of the facilities. The majority of facilities (73.3%) could not accept COVID-19 dialysis patients due to lack of space and manpower. Nosocomial transmission of COVID-19 occurred in 4.0% of the facilities. Of those infected, 51.9% were staff.

**Conclusions:**

This survey revealed that most hemodialysis facilities in Japan had improved implementation of infection control measures and had shortage of PPEs and disinfectants, though some facilities did not implement infection prevention measures adequately, mainly due to the limited space of the facility. It may be recommended that each facility immediately establishes isolation measures to prepare for the pandemic of COVID-19.

**Supplementary Information:**

The online version contains supplementary material available at 10.1186/s41100-021-00350-y.

## Background

Coronavirus disease 2019 (COVID-19), caused by severe acute respiratory syndrome coronavirus 2 (SARS-CoV-2), has become a global pandemic, which has affected a variety of medical treatments such as dialysis [1–3]. The first case of COVID-19 was confirmed in China in November 2019 [4]. In Japan, the first case was reported in January 2020 [5]. COVID-19 rapidly spread across Japan thereafter, as is the case in other countries [6]. Even amidst a pandemic, dialysis facilities need to continuously operate to cater to the constant need of patients who undergo dialysis treatment, three times a week. Infection prevention measures are important in dialysis facilities because patients and staff gather in dialysis and waiting rooms. Moreover, dialysis patients are immunocompromised [7] and are therefore at risk for SARS-CoV-2 infection and its dire complications [8].

However, the infection prevention measures implemented at each dialysis facility in Japan during the pandemic have not yet been investigated on a nationwide scale. In addition, the capacity of each facility to accept dialysis patients with COVID-19 is unknown. Furthermore, several facilities reportedly suffered from a shortage of personal protective equipment (PPE) such as masks and gloves [9, 10]. However, the number of dialysis facilities in Japan that are in similar situations remains unknown. Nosocomial transmission of COVID-19 in dialysis facilities is also a concern. Information regarding these matters should be elucidated, shared, and utilized to derive solutions.

This study aims to conduct a nationwide questionnaire survey regarding the characteristics of facilities, infection prevention measures in routine dialysis practices, shortage of PPE, feasibility of various isolation measures, and nosocomial COVID-19 transmission in the dialysis facilities of the Japanese Association of Dialysis Physicians (JADP) and the Japanese Society for Dialysis Therapy (JSDT) between 20 October and 16 November 2020 (i.e., between the second and third waves in Japan).

## Methods

### Surveyed facilities

The survey was conducted at the member facilities of the JADP and JSDT (n = 4198). These two societies were selected because they were representative of Japanese societies involved with dialysis.

The JADP was established in 1978. Since then, the JADP has been committed to the promotion of appropriate dialysis therapy, improvement of technology, safety and effectiveness, and education and training of healthcare professionals. In addition, the JADP has been promoting measures in the prevention of renal failure and ensuring continuation of dialysis therapy during a disaster [11].

The JSDT was founded in 1968. Since then, the JSDT has contributed to science, research, and dissemination of knowledge on dialysis by conducting research on blood purification, etiology of disease, and pathophysiology, presenting research data, exchanging knowledge, and providing useful information [12].

### Development of the questionnaire

The COVID-19 task force committee was established by the JADP, the JSDT, and the Japanese Society of Nephrology (JSN) [3]. Several authors of the present study were task force committee members. The committee summarized the important points on infection prevention measures for hemodialysis patients during the COVID-19 pandemic. The study content and methodology were then developed and refined. Draft survey instruments were distributed to the members of the committee, and feedback was gathered through e-mail and in-person discussions.

The final agreed upon questionnaire in Japanese and in English are presented in the Additional files 2 and 3. The questionnaire consists of 5 parts, which includes the (i) characteristics of facilities (4 questions), (ii) infection prevention measures in routine hemodialysis practices (20 questions), (iii) shortage of PPE (6 questions), (iv) isolation measures for COVID-19-positive/suspected patients (7 questions), and (v) nosocomial transmission in dialysis units (3 questions). The questions on infection prevention measures were based mainly on the checklist for dialysis treatment from the viewpoint of infection prevention in the Guidelines for Standard Hemodialysis Procedure and Prevention of Infection in Maintenance Hemodialysis Facilities (5th edition) [13], which was developed by the JADP. Since there was no universally accepted definition of nosocomial transmission (healthcare-associated COVID-19) [14], in this study, nosocomial transmission was defined as the horizontal transmission of COVID-19 among staff and patients in a certain facility.

The procedures performed in this study involving human participants were in accordance with the ethical standards of the ethics committee of the JSN (Approval No. 80) and with the 2013 Helsinki Declaration. Informed consent was not necessary because this study was a facility-based survey that did not require information of individual patients. Therefore, the need for written informed consent was waived.

### Data collection

The questionnaires were sent by e-mail from the three related societies and associations (the JADP, JSDT, and JSN) and by mail to eligible facilities. One respondent (such as a doctor, nurse, and medical engineer) from each facility was requested to answer the questionnaire, as a representative of that facility. This questionnaire could be answered anonymously or non-anonymously. In the case of non-anonymous responses, we checked for duplicate responses. If the responses were duplicate, only the first response was considered valid. The response period was between 20 October and 16 November 2020 (i.e., between the second and third waves in Japan). Two methods of response were available, namely faxing the response form or filling out a web form.

### Data analysis

We summarized the responses for descriptive purposes. We compared the implementation status of infection prevention measures before and after the COVID-19 pandemic using the McNemar test. We also compared the implementation status of infection prevention measures, shortage-experiencing rates, and the availability of various isolation measures between hospitals and clinics, using chi-square test. We used the statistical analysis system (SAS University Edition 9.4M7 SAS Institute Inc., Cary, NC) for analysis.

## Results

### Characteristics of respondent facilities

A total of 2310 responses (119 of which were anonymous) were obtained, in which 82 responses were excluded due to duplication from the same facility and one response was excluded because the responding facility was neither a member facility of the JADP nor that of the JSDT, resulting in a total of 2227 valid responses from 2227 facilities.

Among the member facilities of the JADP and the JSDT (N = 4198), 2227 facilities responded, thereby resulting in a 53.0% response rate. The responding facilities were distributed across the country, and there were no apparent differences in the response rates from various regions: Hokkaido 50.7%, Tohoku 54.3%, Kanto (excluding Tokyo) 52.9%, Tokyo 60.0%, Chubu 54.6%, Kinki 48.9%, Chugoku 52.5%, Shikoku 52.8%, Kyusyu 50.6%, and Okinawa 66.2%. Hospitals, which were relatively large facilities that also constituted other departments, accounted for 48.7%, while clinics specialized in dialysis-related services accounted for 50.8%. The remaining respondents (0.4%) answered that they did not belong to either category. This distribution was similar to the 2019 survey results of The JSDT Renal Data Registry (response rate: 98.3%), in which 52.4% of the respondents were hospitals and 47.6% were clinics [15]. Medical facilities designated for infectious diseases accounted for 9.4% of the 2227 responding facilities.

### Implementation of infection prevention measures

The questionnaire asked whether the respondents knew and read the guidelines for standard hemodialysis procedures and prevention of infection in maintenance hemodialysis facilities (5th edition) [13]. Of the respondents, 95.3% stated that they knew the guidelines and 91.3% stated that they had read them.

Table 1 shows the implementation status of each questionnaire item (question numbers in the Appendix were re-numbered from 1 to 17 in Table 1) in the responding dialysis facilities before and during the COVID-19 pandemic. Since all items were included in the guidelines, an implementation rate of 100% was considered desirable.

**Table 1:** Implementation status of infection prevention measures at each dialysis facility, before and after the COVID-19 pandemic occurred

No.	Questions	Implementation rate		*p* value
		Before the pandemic occurred N = 2227 (%)	After the pandemic occurred N = 2227 (%)	
1	Medical instruments for hemodialysis are sterilized or disposable for each patient.	2131 (95.7)	2096 (94.1)	< 0.001
2	Staffs can perform hand hygiene before/after hemodialysis operations, using equipment/supplies in appropriate locations.	2161 (97.0)	2167 (97.3)	0.527
3	Disinfection, maintenance, and inspection of hemodialysis machines are managed according to the instruction manual.	2195 (98.6)	2176 (97.7)	0.003
4	An infection control committee, chaired by the facility manager or the person in charge of nosocomial infection control, has been established and is held regularly with staff from various fields.	1974 (88.6)	1998 (89.7)	0.010
5	Staffs with symptoms of infection such as fever and diarrhea are examined by a doctor whether they can work or not before entering the dialysis room.	1559 (70.0)	2092 (93.9)	< 0.001
6	Priming of the hemodialysis circuit is done just before the treatment with sterile technique in accordance with the package insert.	2091 (93.9)	2090 (93.8)	0.898
7	Initiating and terminating operation are performed with two staffs in a way which does not contaminate with blood.	1306 (58.6)	1313 (59.0)	0.327
8	Staffs always perform careful hand hygiene before and after invasive procedures and wear unused disposable gloves.	2152 (96.6)	2162 (97.1)	0.218
9	Staffs who perform initiating and terminating operation are wearing masks.	1937 (87.0)	2186 (98.2)	< 0.001
10	Staffs who perform initiating and terminating operation are wearing disposable, non-permeable gowns or plastic aprons.	1291 (58.0)	1472 (66.1)	< 0.001
11	Staffs who perform initiating and terminating operation are wearing goggles or face shields.	1145 (51.4)	1648 (74.0)	< 0.001
12	Items contaminated with blood are disposed of as infectious waste or cleaned and sterilized according to the manual.	2195 (98.6)	2177 (97.8)	0.045
13	Heparin and erythropoiesis-stimulating agents are pre-filled syringe products, and other injectable drugs are prepared aseptically in a separate area.	1615 (72.5)	1609 (72.2)	0.343
14	Patients are checked for their temperature and symptoms to confirm that they do not have a suspected infection, before entering the dialysis room.	1199 (53.8)	2095 (94.1)	< 0.001
15	Patients with suspected infection are observed before entering the room, and infection measures are modified according to their conditions.	1601 (71.9)	2141 (96.1)	< 0.001
16	Linens are changed for each patient.	653 (29.3)	765 (34.4)	< 0.001
17	Items that are frequently touched by patient’s and staff’s hands (e.g., doorknobs) are wiped or disinfected several times a day.	1165 (52.3)	2007 (90.1)	< 0.001

During the COVID-19 pandemic, the compliance rates increased significantly in the following eight questions: examination of staff’s physical conditions (No. 5; before 70.0%, during 93.9%, *p* < 0.001); use of PPE, including masks (No. 9; before 87.0%, during 98.2%, *p* < 0.001), disposable, non-permeable gowns or plastic aprons (No. 10; before 58.0%, during 66.1%, *p* < 0.001), and goggles or face shields (No. 11; before 51.4%, during 74.0%, *p* < 0.001) during initiation and termination of hemodialysis; checking the patient’s physical conditions upon entering the dialysis room (No. 14; before 53.8%, during 94.1%, *p*<0.001); modification of infection measures according to each patient’s condition (No. 15; before 71.9%, during 96.1%, *p* < 0.001); bed linen change for each patient (No. 16; before 29.3%, during 34.4%, *p* < 0.001), and disinfection of high frequency contact areas (No. 17; before 52.3%, during 90.1%, *p* < 0.001). However, for the bed linen change for each patient, the implementation rate was only 34.4% even during the pandemic.

The percentages of the other question items did not change significantly before and during the COVID-19 pandemic. The percentages of the items related to hygiene (No. 1, 2, 3, 4, 6, and 8) were around or over 90% before and during the pandemic. However, there were still a few items in which the implementation rate remained relatively low before and during the COVID-19 pandemic. These items included the initiating and terminating operations by two staff members (No. 7; before 58.6%, during 59.0%) and sterile preparation of heparin and erythropoiesis-stimulating agent in a separate area (No. 13; before 72.5%, during 72.2%).

Before the COVID-19 pandemic, 11.1% of the facilities answered that the bed spacing was less than 70 cm; 58.7% answered it was between 70 and 100 cm; and 30.2% answered that it was more than 100 cm. During the pandemic, the corresponding figures were 10.6%, 58.1%, and 31.4%, respectively, suggesting no significant change (*p* = 0.648).

As for the comparison between hospitals and clinics (See Supplementary Table 1, Additional file 1), before the pandemic, there were several items for which the implementation rates were lower in clinics than in hospitals (Nos. 2–4 and 6–17). The implementation rates in clinics improved during the pandemic for some of these items (Nos. 2, 3, 8, 9, 12, and 15), and there were no longer significant differences between the two groups after the pandemic occurred. However, clinics still had significantly lower rates of implementation of other items, including wearing disposable, non-permeable gowns or plastic aprons (No.10; hospitals 82.5%, clinics 50.5%, p < 0.001) and goggles or face shields during initiation and termination of hemodialysis (No. 11; hospitals 82.9%, clinics 65.9%, p < 0.001), even after the pandemic occurred.

### Shortage of PPE under the COVID-19 pandemic

**Fig. 1:**
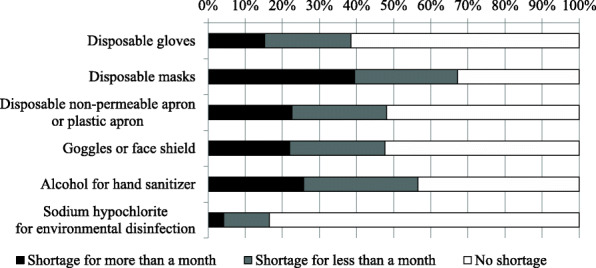
Distribution of the period of shortage of personal protective equipment. The distribution of the period of shortage of six personal infection-protective equipment and disinfectants in dialysis facilities is shown. The black bar indicates shortage for more than a month, the gray bar for less than a month, and the white bar for no shortage

We investigated the duration of shortage of the following PPE during the COVID-19 pandemic: disposable gloves, masks, apron, goggles, face shields, and disinfectants such as alcohol for hand sanitizer and sodium hypochlorite for environmental disinfection (Fig. 1). Notably, 67.2% of the facilities reported a shortage of disposable masks (27.7% for less than a month, 39.5% for more than a month). Alcohol for hand sanitizer was also in short supply in 56.7% of the facilities (30.9% for less than a month, 25.8% for more than a month). There were 222 facilities (10.0%) that experienced a shortage of all the items listed above.

When comparing the shortage of PPE between hospitals and clinics (see Supplementary Table 2, Additional file 1), the shortage rates of disposable masks, and goggles or face shields were higher in hospitals (disposable masks; hospitals 72.3%, clinics 62.6%, p < 0.001, goggles or face shields; hospitals 51.0%, clinics 44.7%, p = 0.003). On the other hand, the shortage rate of alcohol for hand sanitizer was higher in clinics (hospitals 52.9%, clinics 60.3%, p < 0.001).

### Feasibility of various isolation measures against COVID-19-positive and suspected patients

Of all the respondents, 1297 facilities (58.2%) provided hemodialysis for patients suspected of having COVID-19, while 280 facilities (12.6%) provided hemodialysis for COVID-19-positive patients. The number of treated patients per treatment facility ranged from 1 to 20, and the median was 1 (interquartile range 1–2).

**Fig. 2:**
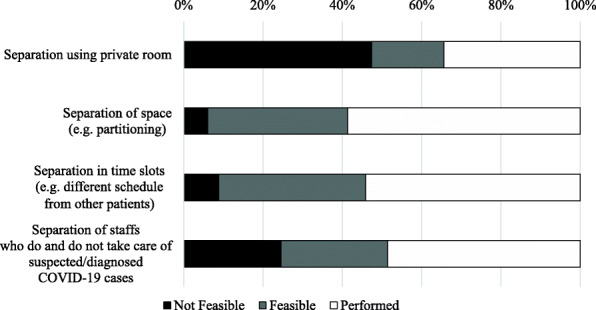
Distribution of the availability of various isolation measures for suspected/diagnosed COVID-19 cases. The distribution of the feasibility of the four isolation measures in dialysis facilities is shown. The black bars indicate the percentage of facilities in which the measure cannot be implemented

The questionnaire also asked about the implementation of four isolation measures for COVID-19-positive/suspected patients (Fig. 2). Separation of space (e.g., partitioning) (93.9%) and separation of time slots (e.g., different schedule from other patients) (91.2%) were available in most facilities (corresponds to the “Feasible” and “Performed” in Fig. 2), while the isolation of COVID-19 patients in a private room was only employed in only 52.7% of the responding facilities. Of the facilities, 75.4% had manpower to separate staff who did and did not take care of suspected/diagnosed COVID-19 cases. On the other hand, 31 facilities (1.4%) responded that they were unable to implement any of the four measures. Comparing hospitals and clinics, there were no significant differences in separation in time slots and separation of staffs who do and do not take care of suspected/diagnosed COVID-19 cases. However, there was a significant difference in separation using private rooms, with 67.6% of respondents in hospitals answering that it was feasible, compared to 38.5% in clinics (p < 0.001, see Supplementary Table 3, Additional file 1).

Among the surveyed facilities, 1632 (73.3%) replied that they could not accept COVID-19-positive patients. The main reason for this was also asked: the majority of the facilities (61.6%) suggested that there was insufficient space for isolation, insufficient manpower (19.9%), little know-how (6.2%), and insufficient infection protection equipment (1.3%). The other facilities (11.0%) answered that they had other reasons that were not specified.

### Nosocomial transmission of COVID-19 in dialysis facilities

**Fig. 3:**
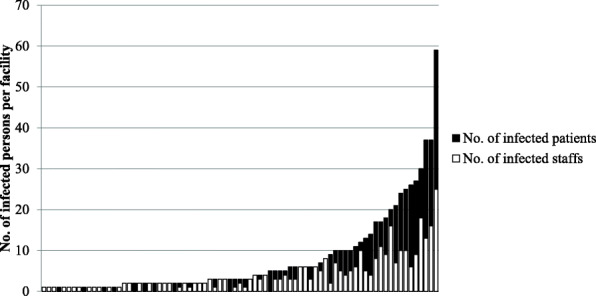
Number of affected patients/staff members in facilities with nosocomial transmission of COVID-19. The number of infected people in the 79 facilities that responded with the details of the number of infected people is shown separately for the staff (white) and for the patients (black). The horizontal axis shows each facility, with a total of 79 bars

A total of 90 facilities (4.0%) reported nosocomial transmission of COVID-19. Of these, 79 facilities responded with the details of the number of infected persons. Figure 3 shows the number of infected staff members and patients in each facility. The number of infected people per hospital ranged from 1 to 59, with a median of 3 (interquartile range 2–10). Of the total number of cases with nosocomial transmissions in all facilities, 51.9% were medical staffs, and the others (48.1%) were patients.

## Discussion

This study is the first nationwide survey involving the member dialysis facilities of the JADP and the JSDT that investigated the implementation of infection control measures. The survey revealed difficulties in infection prevention measures and challenges in the medical care of dialysis patients with COVID-19 during the pandemic mainly due to insufficient space and manpower.

Considering the short time period of this survey (about 1 month), we consider that a response rate of 53% is relatively high in this type of urgent surveys. However, we acknowledge that it is lower than that of the annual survey in the JSDT Renal Data Registry with a response rate of around 98% [15]. In the context of the COVID-19 pandemic, we prioritized to report the results as quickly as possible, instead of making a further action to increasing the response rate.

The Guidelines for Standard Hemodialysis Procedure and Prevention of Infection in Maintenance Hemodialysis Facilities (5th edition) [13] were well known to the vast majority of the dialysis facilities that responded to this survey (95.3%). With regards to the questions about maintenance of hemodialysis equipment, hand hygiene, and appropriate disposal of blood contaminants, more than 90% of the facilities reported that they were continuously implementing these measures before and during the COVID-19 pandemic. Meanwhile, the compliance rates of the staff and patients, donning of masks during the initiation and termination of hemodialysis operation, and disinfection of frequently contacted areas showed significant improvements during the COVID-19 pandemic, reaching over 90% compliance rates. The use of disposable, non-permeable gowns, plastic aprons, goggles, or face shields when initiating and terminating hemodialysis operation was also significantly improved; however, the implementation rate remained relatively low (66.1% and 74.0%, respectively). Regardless of the COVID-19 pandemic, it is generally recommended that PPE be worn during the initiation and termination of hemodialysis operations to prevent infection, as blood splashes may occur during these procedures.

Regarding the question “Initiation and termination of operation are performed with two staff members in a way that does not contaminate with blood.” (No. 7 in Table 1), the guidelines state that it was preferable to have one person on the patient side and another person on the machine side when initiating hemodialysis (i.e., when connecting the puncture needle to the blood circuit) who would work together. In terminating hemodialysis (i.e., in returning blood to the patient), it was preferable to have two staff members working together.

With regard to the item about the preparation of ESA and heparin (No. 13 in Table 1), the implementation rate was approximately 72%. There was no significant difference before and during the COVID-19 pandemic. Zoning of these preparations so that there is no crossover of pre-/post-use medicine was important not only for preventing the spread of COVID-19 but also for the prevention of various infectious diseases. A higher implementation rate was desirable.

Although the guidelines developed by the JADP recommend changing linens for each patient (No. 16 in Table 1), only 34.4% of the facilities had been implementing this even during the COVID-19 pandemic. However, the guidelines also stated that the practice depended on the situation of each facility, as changing of linens might cause dust to fly around and contaminate the environment or might be inhaled by patients.

Similarly, although a bed spacing of 100 cm or more was recommended, only about 30% of the facilities were able to adapt this. A considerable number of facilities answered that the spacing was less than 70 cm. Although this might be unavoidable to some extent due to the small geographical area of Japan and the fact that there were few facilities with large areas, it was clear that maintaining a social distance was important to prevent the spread of COVID-19. Ideally, the length between beds should at least be 100 cm.

With regard to the shortage of PPE and disinfectants (Fig. 1), more than 50% of the facilities were running short of masks and alcohol for hand sanitizers due to the COVID-19 pandemic. It was important to be equipped with adequate supplies.

Regarding the number of hemodialysis patients with COVID-19 that could be accepted at each facility, 73.3% of the facilities answered 0. The most common reason for this was the lack of an isolation space. Not many facilities had private rooms, and only 52.7% reported that they could implement isolation measures using private rooms (Fig. 2). These phenomena may also be attributed to the small number of spacious facilities, as mentioned earlier. To compensate for this shortcoming, it was important for each region to organize the flow of referring COVID-19 patients to hospitals that could accept them. In addition, although the number was small, there were facilities that could not implement any of the four isolation measures. These facilities were strongly urged to establish isolation measures as soon as possible.

Our comparison between hospitals and clinics showed that clinics had lower rates of implementation of infection prevention measures, and fewer facilities were able to provide separation using private rooms, even after the pandemic occurred. This may be because clinics generally have fewer human resources and smaller spaces than hospitals. Meanwhile, the lower implementation rates of wearing disposable, non-permeable gowns or plastic aprons, and goggles or face shields in clinics seem to be modifiable and need to be improved as soon as possible. The shortage rates of PPE also differed between clinics and hospitals, and this may be due to the differences in the distribution channels and size of the storage space.

In the facilities where nosocomial transmission of COVID-19 occurred, the staff accounted for almost half (51.9%) of the cases. Thus, nosocomial transmission not only has a direct negative consequence on affected patients but could also greatly reduce the number of medical staff and the quality of medical care for non-affected patients (Fig. 3).

This study has several limitations. For instance, the response rate of the survey was 53.0%. Although the response rate did not vary widely among different regions in Japan, the representativeness of the facilities participating in the survey remained unknown in other aspects. For example, facilities responding to the questionnaire might be better equipped and prepared for the COVID-19 pandemic than other facilities. In addition, there might be recall bias or misreporting in the questionnaire, which might have led to misclassification of the results.

Finally, this survey was conducted from October to November 2020, during a period between the “second wave” and “third wave” in Japan. The infection control measures may have changed thereafter.

## Conclusions

This nationwide survey revealed that dialysis facilities treated general hemodialysis patients with COVID-19 by using various infection control strategies. Some facilities were unable to implement infection prevention measures adequately, mainly due to the limited space of the facility, lack of manpower, and temporal shortage of PPE. Since nosocomial infections had a negative impact on patients, staff, and hospital management, it was recommended that each facility immediately establish isolation measures to prevent nosocomial infections and to prepare for the further spread of COVID-19 and the emergence of other infectious diseases in the future.

## Supplementary Information

**Additional file 1: Supplementary Table 1**.Implementation status of infection prevention measures at each dialysis facility, before and after the COVID-19 pandemic occurred, by facility type. **Supplementary Table 2**. Percentages experiencing shortages of personal protective equipment due to pandemic, by facility type. Supplementary Table 3. Percentages of the availability of various isolation measures for suspected/diagnosed COVID-19 cases, by facility type.

**Additional file 2:.** Dialysis Questionnaire Original (Japanese).

**Additional file 3:.** Dialysis Questionnaire English Translation.

## Data Availability

The datasets used and/or analyzed in the current study are available from the corresponding author upon reasonable request.

## Abbreviations

COVID-19: Coronavirus disease 2019
SARS-CoV-2: Severe acute respiratory syndrome coronavirus 2
JADP: Japanese Association of Dialysis Physicians
JSDT: Japanese Society for Dialysis Therapy
JSN: Japanese Society of Nephrology
PPE: Personal protective equipment
