# Supplementary material for: Infection prevention measures for patients undergoing hemodialysis during the COVID-19 pandemic in Japan: a nationwide questionnaire survey
Source: Ren Replace Ther. 2021 May 29;7(1):27. doi: 10.1186/s41100-021-00350-y (PMC8164066; doi:10.1186/s41100-021-00350-y)
Supplement: Supplementary file 1 — Additional file 1: Supplementary Table 1.Implementation status of infection prevention measures at each dialysis facility, before and after the COVID-19 pandemic occurred, by facility type. Supplementary Table 2. Percentages experiencing shortages of personal protective equipment due to pandemic, by facility type. Supplementary Table 3. Percentages of the availability of various isolation measures for suspected/diagnosed COVID-19 cases, by facility type. [file 41100_2021_350_MOESM1_ESM.docx]

Supplementary table 1. Implementation status of infection prevention measures at each dialysis facility, before and after the COVID-19 pandemic occurred, by facility type.

|  |  | Before the pandemic occurred | | | | | After the pandemic occurred | | | | | |
| --- | --- | --- | --- | --- | --- | --- | --- | --- | --- | --- | --- | --- |
| No. | Questions | **Hospitals**  N=1,085 (%) | | **Clinics**  N=1,132 (%) | | *p*  value | **Hospitals**  N=1,085 (%) | | | **Clinics**  N=1,132 (%) | | *p*  value |
| 1 | Medical instruments for hemodialysis are sterilized or disposable for each patient. | 1,039 (95.8) | 1,082 (95.6) | | 0.838 | | | 1,018 (93.8) | 1,068 (94.4) | | 0.603 | |
| 2 | Staffs can perform hand hygiene before/after hemodialysis operations, using equipment/supplies in appropriate locations. | 1,069 (98.5) | 1,082 (95.6) | | <0.001 | | | 1,051 (96.9) | 1,106 (97.7) | | 0.225 | |
| 3 | Disinfection, maintenance and inspection of hemodialysis machines are managed according to the instruction manual. | 1,075 (99.1) | 1,110 (98.1) | | 0.044 | | | 1,063 (98.0) | 1,103 (97.4) | | 0.402 | |
| 4 | An infection control committee, chaired by the facility manager or the person in charge of nosocomial infection control, has been established and is held regularly with staff from various fields. | 1,062 (97.9) | 906 (80.0) | | <0.001 | | | 1,055 (97.2) | 935 (82.6) | | <0.001 | |
| 5 | Staffs with symptoms of infection such as fever and diarrhea are examined by a doctor whether they can work or not before entering the dialysis room. | 752 (69.3) | 800 (70.7) | | 0.484 | | | 1,009 (93.0) | 1,074 (94.9) | | 0.063 | |
| 6 | Priming of the hemodialysis circuit is done just before the treatment with sterile technique in accordance with the package insert. | 1,042 (96.0) | 1,042 (92.1) | | <0.001 | | | 1,031 (95.0) | 1,051 (92.8) | | 0.032 | |
| 7 | Initiating and terminating operation are performed with two staffs in a way which does not contaminate with blood. | 714 (65.8) | 584 (51.6) | | <0.001 | | | 713 (65.7) | 592 (52.3) | | <0.001 | |
| 8 | Staffs always perform careful hand hygiene before and after invasive procedures and wear unused disposable gloves. | 1,061 (97.8) | 1,081 (95.5) | | 0.003 | | | 1,058 (97.5) | 1,094 (96.6) | | 0.226 | |
| 9 | Staffs who perform initiating and terminating operation are wearing masks. | 1,002 (92.4) | 928 (82.0) | | <0.001 | | | 1,066 (98.3) | 1,110 (98.1) | | 0.737 | |
| 10 | Staffs who perform initiating and terminating operation are wearing disposable, non-permeable gowns or plastic aprons. | 848 (78.2) | 440 (38.9) | | <0.001 | | | 895 (82.5) | 572 (50.5) | | <0.001 | |
| 11 | Staffs who perform initiating and terminating operation are wearing goggles or face shields. | 750 (69.1) | 394 (34.8) | | <0.001 | | | 899 (82.9) | 746 (65.9) | | <0.001 | |
| 12 | Items contaminated with blood are disposed of as infectious waste or cleaned and sterilized according to the manual. | 1,078 (99.4) | 1,107 (97.8) | | 0.002 | | | 1,066 (98.3) | 1,101 (97.3) | | 0.118 | |
| 13 | Heparin and erythropoiesis-stimulating agents are pre-filled syringe products, and other injectable drugs are prepared aseptically in a separate area. | 811 (74.8) | 798 (70.5) | | 0.025 | | | 808 (74.5) | 794 (70.1) | | 0.023 | |
| 14 | Patients are checked for their temperature and symptoms to confirm that they do not have a suspected infection, before entering the dialysis room. | 634 (58.4) | 557 (49.2) | | <0.001 | | | 1,039 (95.8) | 1,046 (92.4) | | 0.001 | |
| 15 | Patients with suspected infection are observed before entering the room, and infection measures are modified according to their conditions. | 824 (75.9) | 770 (68.0) | | <0.001 | | | 1,045 (96.3) | 1,086 (95.9) | | 0.646 | |
| 16 | Linens are changed for each patient. | 383 (35.3) | 269 (23.8) | | <0.001 | | | 434 (40.0) | 329 (29.1) | | <0.001 | |
| 17 | Items that are frequently touched by patient’s and staff’s hands (e.g., doorknobs) are wiped or disinfected several times a day. | 625 (57.6) | 533 (47.1) | | <0.001 | | | 957 (88.2) | 1,041 (92.0) | | 0.003 | |
| 18 | Bed spacing more than 100 cm. | 439 (40.5) | 231 (20.4) | | <0.001 | | | 452 (41.7) | 244 (21.6) | | <0.001 | |

Supplementary table 2. Percentages experiencing shortages of personal protective equipment due to pandemics, by facility type.

|  | Hospitals  N=1,085  (%) | Clinics  N=1,132 (%) | *p*  value |
| --- | --- | --- | --- |
| Disposable gloves | 417 (38.4) | 440 (38.9) | 0.833 |
| Disposable masks | 784 (72.3) | 709 (62.6) | <0.001 |
| Disposable non-permeable apron or plastic apron | 522 (48.1) | 547 (48.3) | 0.921 |
| Goggles or face shield | 553 (51.0) | 506 (44.7) | 0.003 |
| Alcohol for hand sanitizer | 574 (52.9) | 683 (60.3) | <0.001 |
| Sodium hypochlorite for environmental disinfection | 193 (17.8) | 174 (15.4) | 0.126 |

Supplementary table 3. Percentages of the availability of various isolation measures for suspected/diagnosed COVID-19 cases, by facility type.

|  | Hospitals  N=1,085  (%) | Clinics  N=1,132  (%) | *p*  value |
| --- | --- | --- | --- |
| Separation using private room | 733 (67.6) | 436 (38.5) | <0.001 |
| Separation of space (e.g. partitioning) | 1,000 (92.2) | 1,083 (95.7) | 0.001 |
| Separation in time slots  (e.g. different schedule from other patients) | 985 (90.8) | 1,034 (91.3) | 0.644 |
| Separation of staffs who do and do not take care of suspected/diagnosed COVID-19 cases | 801 (73.8) | 872 (77.0) | 0.079 |
