# Supplementary material for: Infection prevention measures for patients undergoing hemodialysis during the COVID-19 pandemic in Japan: a nationwide questionnaire survey
Source: Ren Replace Ther. 2021 May 29;7(1):27. doi: 10.1186/s41100-021-00350-y (PMC8164066; doi:10.1186/s41100-021-00350-y)
Supplement: Supplementary file 2 — Additional file 2:. Dialysis Questionnaire Original (Japanese). [file 41100_2021_350_MOESM2_ESM.pdf]

【血液透析患者の COVID-19 予防・診療体制調査】

【1】貴施設が当てはまるものをお選びください。

☐　日本透析医会会員施設

☐　日本透析医学会会員施設

☐　日本腎臓学会認定教育施設

【2】貴施設は感染症指定病院ですか。

☐　はい

☐　いいえ

【3】貴施設が当てはまるものはどれですか。

☐　病院

☐　透析クリニック（有床診療所）

☐　透析クリニック（無床診療所）

☐　その他（　　　　　　　　　　　　　　　　　　　　　　　　）

【4】1 日に平均何人の透析を行っていますか。

（　　　　　　　　　）　人

＜透析室での感染予防対策について＞

貴施設の透析部署で行っている感染予防対策に☑をつけてください。

（COVID-19 流行前・流行後のいずれも実施している場合は、

両方に☑をつけてください。）

	感染予防対策	COVID-19 流行前	COVID-19 流行後
【5】	透析に使用する医療器具は患者ごとに滅菌されている、あるいはディスポーザブルである	☐	☐
【6】	スタッフが透析操作前後に手指衛生（手洗い、アルコール製剤による消毒など）を容易にできる設備・物品が適切な場所にある	☐	☐
【7】	ベッド間隔につき当てはまるものを選んでください＊	☐　70cm 未満<br>☐　70～100cm<br>☐　100cm 以上	☐　70cm 未満<br>☐　70～100cm<br>☐　100cm 以上

	感染予防対策	COVID-19 流行前	COVID-19 流行後
【8】	透析装置の消毒や保守点検は取り扱い説明書に従い管理されている	□	□
【9】	施設管理責任者あるいは院内感染対策担当者を委員長とした感染対策委員会が設置され、各職種のスタッフが参加して定期的に開催されている	□	□
【10】	スタッフに発熱や下痢等の感染症を疑う症状のある時は透析室に入室する前に医師の診察を受け就業可能か指示を仰いでいる	□	□
【11】	透析回路のプライミングは治療直前に、手指衛生を行い清潔操作で添付文書に基づいた方法で行っている	□	□
【12】	穿刺・回収を二人で行うなど機械を血液汚染させない方法で行っている	□	□
【13】	スタッフは侵襲的手技の前後に入念な手指衛生を必ず行い、未使用のディスポーザブル手袋を装着している	□	□
【14】	穿刺および抜針操作をするスタッフは、マスクを装着している	□	□
【15】	穿刺および抜針操作をするスタッフは、ディスポーザブルの非透水性ガウンまたはプラスチックエプロンを装着している	□	□
【16】	穿刺および抜針操作をするスタッフは、ゴーグルあるいはフェイスシールドを装着している	□	□

	感染予防対策	COVID-19 流行前	COVID-19 流行後
【17】	血液に汚染された物品は周囲を汚染しないように注意して感染性廃棄物として廃棄するか、マニュアルにのっとり洗浄滅菌されている	□	□
【18】	ヘパリンや ESA 製剤はプレフィルドシリンジ製品を使用し、それ以外の透析中に投与される注射薬剤は、透析室から区画された場所で無菌的に準備されている	□	□
【19】	患者が感染症が疑われる状態にないかどうか、体温測定・症状の有無の確認などを用いて、入室前に確認している	□	□
【20】	感染症の疑われる患者を入室前に観察し、状態にあわせて対策を変更している	□	□
【21】	リネン類は患者ごとに交換している	□	□
【22】	患者から離れた場所で患者やスタッフの手指が高頻度に接触する場所（ドアノブ等）は 1 日数回清拭や消毒を行っている	□	□

＜感染防護具等の準備状況と不足状況について＞
貴施設の不足状況を教えてください。（COVID-19 流行後）

	品名	1 ヶ月以上不足した	1 ヶ月未満だが不足した	常に充足していた
【23】	ディスポーザブル手袋	□	□	□
【24】	マスク	□	□	□
【25】	ディスポーザブル非透水性エプロンあるいはプラスチックエプロン	□	□	□
【26】	ゴーグルあるいはフェイスシールド	□	□	□
【27】	手指消毒用アルコール	□	□	□
【28】	次亜塩素酸ナトリウム（環境消毒用）	□	□	□

＜透析施設における標準的な透析操作と感染予防に関するガイドラインについて＞
【29】上記ガイドライン 5 訂版をご存知ですか。
□ はい
□ いいえ

【30】上記ガイドライン 5 訂版を読んだことはありますか。
□ はい
□ いいえ

＜COVID-19 症例の診療経験について＞
【31】COVID-19 が疑われた透析症例の診療経験はありますか。
□ はい
□ いいえ

【32】COVID-19 の確定診断がなされた透析症例は貴施設では何例経験されましたか。
(　　　　　　　　　　) 例

COVID-19 疑い例/診断例の透析の際に、以下の措置を行うことは可能ですか、実際に実施しましたか。

【33】個室隔離透析 ＊

	はい	いいえ
可能である	□	□
実施した	□	□

【34】個室は最大何室用意できますか？

（　　　　　　　　　）　室

【35】空間的隔離（パーティションで区切るなど） ＊

	はい	いいえ
可能である	□	□
実施した	□	□

【36】時間的隔離（他患者と時間をずらすなど） ＊

	はい	いいえ
可能である	□	□
実施した	□	□

【37】対応するスタッフを分ける ＊

	はい	いいえ
可能である	□	□
実施した	□	□

【38】PCR 検査あるいは抗原検査が必要と医師が判断してから、受けるまでに何日を要しましたか。（自由回答）

※「検査を要した症例はいなかった」場合は「-999」とご入力願います※

（　　　　　　　　　）　日

【39】PCR 検査あるいは抗原検査は自施設内で行うことが可能でしたか。

□　はい

□　いいえ

【40】転入院が必要な COVID-19 に罹患した透析症例について、転入院が必要と判明してから実際に転院するまで、平均で何日を要しましたか。（自由回答）
※「転院を要した症例はいなかった」場合は「-999」とご入力願います※
(　　　　　　　　　)　日

【41】COVID-19 に罹患した透析患者を最大何人まで受け入れ可能ですか。
(　　　　　　　　　)　人

【42】これ以上の受け入れを阻む要因のうち最も影響が大きいものはどれですか。
□　人手が足りない
□　隔離するスペースがない
□　感染防護具が足りない
□　対応するノウハウがない
□　その他

【43】COVID-19 の院内感染（院内におけるスタッフあるいは患者の水平感染）は起こりましたか。
□　はい
□　いいえ

【44】起きた場合、計何人が感染しましたか。
(　　　　　　　　　)　人

【45】そのうち、スタッフは何人ですか。
(　　　　　　　　　)　人

【46】COVID-19 予防対策、診療体制において、工夫した点、困った点、その他お気づきの点があれば、ぜひ教えてください。
