# Supplementary material for: Infection prevention measures for patients undergoing hemodialysis during the COVID-19 pandemic in Japan: a nationwide questionnaire survey
Source: Ren Replace Ther. 2021 May 29;7(1):27. doi: 10.1186/s41100-021-00350-y (PMC8164066; doi:10.1186/s41100-021-00350-y)
Supplement: Supplementary file 3 — Additional file 3:. Dialysis Questionnaire English Translation. [file 41100_2021_350_MOESM3_ESM.pdf]

Survey of COVID-19 prevention and treatment systems for hemodialysis patients
(English translation)

Questions:

[1] Please select all that applies to your facility.
□ Member facility of the Japanese Association of Dialysis Physicians (JADP)
□ Member facility of the Japanese Society of Dialysis Therapy (JSDT)
□ Certified educational facility of the Japanese Society of Nephrology (JSN)

[2] Is your facility a designated hospital for infectious diseases?
□ Yes
□ No

[3] Which of the following applies to your facility?
□ Hospital
□ Dialysis clinic with inpatient beds
□ Dialysis clinic without inpatient beds
□ Other

[4] On average, how many patients do you treat with dialysis per day?
(   ) persons

< Infection prevention measures in the dialysis room>

Please select the infection prevention measures you are taking in your dialysis department.
If they have been implemented both before and after the COVID-19 pandemic, please check both boxes.

No.	Infection prevention measures	Before the start of the pandemic	During the pandemic
[5]	Medical instruments for hemodialysis are sterilized or disposable for each patient.	□	□
[6]	Staff members perform hand hygiene before/after hemodialysis operations, using equipment/supplies at appropriate locations.	□	□
[7]	Please select the applicable bed spacing in your facility.	□<70 cm<br>□70-100 cm<br>□>100 cm	□<70 cm<br>□70-100 cm<br>□>100 cm

No.	Infection prevention measures	Before the start of the pandemic	During the pandemic
[8]	Disinfection, maintenance and inspection of hemodialysis machines are managed according to the instruction manual.	□	□
[9]	An infection control committee, chaired by the facility manager or the person in charge of nosocomial infection control, has been established and is held regularly with staff from various fields.	□	□
[10]	Staff members with symptoms of infection such as fever and diarrhea are examined by a doctor to determine whether they can work before entering the dialysis room.	□	□
[11]	Priming of the hemodialysis circuit is performed just before treatment with a sterile technique in accordance with the package insert.	□	□
[12]	Initiation and termination of operation are performed with two staff members in a way that does not contaminate with blood.	□	□
[13]	Staff members always perform careful hand hygiene before and after invasive procedures and wear unused disposable gloves.	□	□
[14]	Staff members who initiate and terminate operation wear masks.	□	□
[15]	Staff members who initiate and terminate operation wear disposable, non-permeable gowns or plastic aprons.	□	□
[16]	Staff members who initiate and terminate operation wear goggles or face shields.	□	□
[17]	Items contaminated with blood are disposed of as infectious waste or cleaned and sterilized according to the manual.	□	□
[18]	Heparin and erythropoiesis stimulating agents are pre-filled syringe products, and other injectable drugs are prepared aseptically in a separate area.	□	□
[19]	Patients are checked for their temperature and symptoms to confirm that they do not have a suspected infection before entering the dialysis room.	□	□

No.	Infection prevention measures	Before the start of the pandemic	During the pandemic
[20]	Patients with suspected infection are observed before entering the room, and infection measures are modified according to their conditions.	□	□
[21]	Bed linen is changed for each patient.	□	□
[22]	Items that are frequently touched by patients' and staff's hands (e.g., doorknobs) are wiped or disinfected several times a day.	□	□

＜ Status of preparation and shortage of personal protective equipment

Please tell us the shortage situation at your facility during the COVID-19 epidemic.

No.	Item	Shortage for more than a month	Shortage for less than a month	No shortage
[23]	Disposable gloves	□	□	□
[24]	Disposable masks	□	□	□
[25]	Disposable non-permeable apron or plastic apron	□	□	□
[26]	Goggles or face shield	□	□	□
[27]	Alcohol for hand sanitizer	□	□	□
[28]	Sodium hypochlorite for environmental disinfection	□	□	□

＜ Guidelines for standard hemodialysis procedures and prevention of infection in maintenance of hemodialysis facilities[13] ＞

[29] Did you know that the 5th edition of the above guideline has been published?

□ Yes

□ No

[30] Have you read the 5th edition of the above guidelines?

□uYes

□eNo

＜Experience in treating patients with COVID-19 positive/suspected dialysis＞

[31] Do you have any experience treating dialysis patients suspected of having COVID-19?

□rYes

□eNo

[32] How many dialysis cases with a confirmed diagnosis of COVID-19 have you encountered at your facility?

(　) cases

For dialysis of suspected/diagnosed COVID-19 cases, it is possible to implement the following measures. Have you implemented the following measures?

[33] Separation using private rooms

	Yes	No
Feasible	□	□
Performed	□	□

[34] How many private rooms can be prepared?

(　) rooms

[35] Separation of space (e.g., partitioning)

	Yes	No
Feasible	□	□
Performed	□	□

[36] Separation in time slots (e.g., different schedules from other patients)

	Yes	No
Feasible	□	□
Performed	□	□

[37] Separation of staff who do and do not take care of suspected/diagnosed COVID-19 cases

	Yes	No
Feasible	□	□
Performed	□	□

[38] How many days did it take from the time the doctor determined that the patient needed PCR or antigen testing until the patient received it? Please enter "-999" if there were no cases that required testing.

(　) days

[39] Can PCR test or antigen test be performed at your facility?

□yYes

□eNo

[40] For dialysis patients with COVID-19 requiring hospital transfer, how many days on average did it take from the time when it was determined that the patient needed to be transferred until when the patient was actually transferred? Please enter "-999" if there were no cases that required transfer.
(   ) days

[41] What is the maximum number of dialysis patients affected by COVID-19 that your facility can accept?
(   ) persons

[42] Which of the following factors would have the greatest impact on preventing you from accepting any more dialysis patients with COVID-19?
□ Lack of manpower
□ Lack of space for isolation
□ Lack of personal protective equipment
□ Lack of know-how to deal with the situation
□ Other

[43] Has nosocomial transmission (horizontal transmission of COVID-19 infection among staff or patients in the facility) of COVID-19 occurred in your facility?
□yYes
□eNo

[44] If this happened, how many people were infected in total?
(   ) persons

[45] How many of them were staff members?
(   ) persons

[46] Please tell us about any innovations, difficulties, or other points you have observed in your COVID-19 prevention measures and medical treatment system.
(   )
